# Prevalence of Internet Gaming Disorder and Associated Factors Among High School Students in Zawiya City, Libya: A Cross-Sectional Study

**DOI:** 10.7759/cureus.107471

**Published:** 2026-04-21

**Authors:** Rehab A Sherlala, Wasan A Rawag, Sarah J Elfurdag, Ziyad F Alatrash, Zinab Elfituri, Entisar K Aboukanda

**Affiliations:** 1 Family and Community Medicine, Faculty of Medicine, University of Zawia, Zawia, LBY; 2 Medicine, Faculty of Medicine, University of Tripoli, Tripoli, LBY; 3 Medicine, Faculty of Medicine, University of Zawia, Zawia, LBY; 4 Medical Physiology, Faculty of Medicine, University of Zawia, Zawia, LBY

**Keywords:** academic performance, anxiety, depression, high school, internet gaming disorder, libya

## Abstract

Internet Gaming Disorder (IGD) has emerged as a significant public health concern globally and is increasingly recognized as a behavioral condition affecting adolescents. This study aimed to estimate the prevalence of IGD and identify its association with sociodemographic, academic, behavioral, and psychological factors among high school students in Zawiya City, Libya.

This cross-sectional study employed multistage cluster sampling, randomly selecting eight public high schools in Zawiya City, from which a total of 369 students were recruited. Data were collected between November 2024 and January 2025 using an interviewer-administered questionnaire that included sociodemographic characteristics, gaming-related behaviors, the nine-item Internet Gaming Disorder Scale-Short Form (IGDS9-SF), the Patient Health Questionnaire-9 (PHQ-9), and the Generalized Anxiety Disorder-7 (GAD-7) scale. Chi-square and t-tests were applied, with statistical significance set at *P* ≤ 0.05.

Among 369 high school students, 50.4% were male and 43.1% were in 10th grade. Overall, 66.4% of students reported engaging in video gaming, of whom 62.4% were male. The prevalence of IGD was 7.3% among gamers (245 students), with a mean IGD score of 18.6. Furthermore, students with IGD demonstrated lower school attendance and lower grade point averages. More than half of gamers with IGD experienced moderate to severe anxiety, while 28% exhibited moderately severe to severe depression.

The high prevalence of anxiety and depression, and IGD among high school gamers in Zawiya City, Libya, represents a significant public health concern. These findings highlight the need for additional studies to better identify underlying causes and to guide the development of targeted prevention strategies and effective educational interventions.

## Introduction

Internet Gaming Disorder (IGD) is a growing public health concern in the digital age. The World Health Organization in the 11th revision of the International Classification of Diseases (ICD-11) reported IGD as a form of gaming (digital or video) behavior, which is characterized by an inability to govern gaming activities, an attribution of a greater priority to gaming than other activities, and continuation of gaming or an increase in time spent in gaming even when there are negative consequences [1]. Additionally, within the recently updated Diagnostic and Statistical Manual of Mental Disorders, Fifth Edition (DSM-5), the American Psychiatric Association (APA) introduced IGD in Section III as a new condition for further research; it is defined as an individual’s persistent and recurrent use of the internet for gaming to the point that it leads to either personal or social health issues [2].

Globally, the prevalence of IGD has increased in parallel with the growth of digital technology [1,3]. In a meta-analysis including 53 studies with participants across 17 different countries, Stevens et al. found that the pooled IGD prevalence was 3.05%; a significantly higher prevalence was found to be 4.6% among adolescent participants [4]. Research reports that IGD has significant psychological, social, and academic consequences, influenced by cultural and socio-economic factors, which in particular play out among adolescents and young adults [4]. Also, long-term gaming periods and lack of being able to control gaming activity are potential risk factors for IGD, reflecting the concept of tolerance within its definition [5]. Additionally, it is also noted that male gender, disrupted sleep pattern, prolonged screen exposure, and excessive gaming in leisure time are significant predictors of IGD [5,6]. However, other studies suggested that females may also be at risk, which is still a matter of debate [4,5]. Beyond the behavioral issues, it is suggested that IGD is associated with poor mental health and well-being, which includes stress, sleep disturbance, social isolation, obesity, depression, and anxiety [6,7]. In addition, studies have shown that IGD is associated with a decrease in academic performance, as well as employment problems, which underscore its broader economic and social impact [7]. Given these major concerns, it is reported that countries like China, Japan, and South Korea have imposed gaming regulations and implemented public health programs to reduce excessive use of gaming, while worldwide, many studies have been carried out on the prevalence and related factors of IGD in different populations [4,7].

In Libya, internet penetration has reached 88.4%, and digital gaming has emerged as an increasingly popular recreational activity, according to the 2024 Digital Libya report [8]. However, empirical research addressing IGD and its impact on young people within the Libyan context remains very limited despite the increasing global recognition of IGD as a behavioral condition affecting adolescents. To date, no published studies have comprehensively examined the prevalence and risk factor correlates of IGD among high school students in Libya. One cross-sectional study has explored online gaming addiction during the COVID-19 pandemic, and Libyan participants accounted for just 1% of the total study sample, substantially limiting the generalizability of its findings to the Libyan population [9]. This lack of regional evidence limits the development of culturally appropriate prevention and intervention strategies.

This study addresses a major research gap in the literature by examining the prevalence and related factors of IGD among adolescents in Libya, a region where such research is critically limited. Specifically, this study aims to estimate the prevalence of IGD among high school students in Zawiya City, Libya, and to examine the association between IGD and sociodemographic, behavioral, academic, and psychological factors.

## Materials and methods

Study design and setting

A descriptive cross-sectional study was conducted to assess the prevalence of IGD and its association with sociodemographic, academic, behavioral, and psychological (depression and anxiety) factors among public high school students in Zawiya City, which is located in western Libya. The city includes 28 public high schools (at the time of data collection) that are geographically distributed throughout the city, serving students in grades 10th, 11th, and 12th. Data was collected inside the classrooms of the selected schools between November 2024 and January 2025.

Target population and study sampling

The study population consisted of all students enrolled in public high schools in Zawiya City during the 2024-2025 academic year. A list of all public high schools in Zawiya City was obtained from the Directorate of Education and served as the sampling frame. The total number of students in the target population was 7,520, distributed across 28 public high schools. Students between the ages of 15 and 17, of both genders, from all academic grades (10th, 11th, 12th), and attending public high schools of the central Zawiya school districts were eligible for inclusion in the study. Students who did not meet these criteria were excluded.

A multistage cluster sampling technique was applied to obtain a representative sample of public high school students in Zawiya City, Libya. The sampling process was conducted in three stages to ensure adequate representation across schools and academic grades.

First Stage - School Selection

All 28 public high schools in Zawiya were listed and assigned unique identification numbers. Using a simple random sampling technique with a random number generation process, eight schools were selected. These schools were geographically distributed across different districts of the city, ensuring representativeness of the wider student population.

Second Stage - Class Selection

In each of the eight schools selected, classes were initially stratified first by academic grade (10th, 11th, and 12th grades) to ensure proper presentation. Within each grade, classes were randomly selected using simple random sampling based on a computer-generated list of random numbers provided by the school administration. One or two classes were selected at random from each grade, with distribution of the selected classes roughly proportional to the total number of classes in each grade, typically two or three per grade. This approach ensured a balance between adequate grade representation and the practical goal of achieving the target sample size. Moreover, since students in Libyan public schools within the same grade are generally homogeneous in terms of age, curriculum, and socioeconomic background, entire classes were considered suitable and representative sampling clusters for the study.

Third Stage - Student Inclusion

All students in the selected classes were invited to participate in the study. Prior to data collection, the research team explained the study objectives and procedures to students, emphasizing voluntary participation and confidentiality. Students who refused participation or were absent on the day of data collection were excluded without replacement; however, refusals were minimal (<3%) and did not meaningfully affect the final sample size. By including all students in the randomly selected classes, the study minimized selection bias and ensured representativeness across all academic grades and schools.

Sample size determination

The initial sample size was determined using a single population proportion formula, considering a 5% margin of error, 95% confidence interval, a total population size of 7,520 students, and an estimated prevalence of 21.9% based on previous studies. The formula used was *n *= *Z*^2 ^×* p *(1 -* p*)/*d*^2^,where *n *is the required sample size, *Z *is the standard normal value corresponding to a 95% confidence level, *p *is the estimated prevalence, and *d *is the margin of error. Based on this calculation, the minimum required sample size was approximately 263 participants. Since the present study employed a multistage cluster sampling design, a design effect (DEFF) of 1.5 was applied to account for the potential loss of precision due to clustering at the class and school levels. This value was selected with recommendations from previous cluster survey methodologies conducted in similar settings, where DEFF values between 1.2 and 2.0 were commonly adopted in the absence of prior intraclass correlation estimates [10,11]. Thus, the adjusted sample size was calculated as follows: *n*_(adjusted)_ = 263 × 1.5 = 395. Therefore, the target sample size was 395 students.

After data collection and quality review, incomplete questionnaires were excluded from the analysis if more than 20% of key items, particularly those pertaining to the IGD, anxiety, or depression scale, were left unanswered. This exclusion criterion was applied to ensure data quality and maintain the validity of scale-derived scores, as excessive missing responses could bias total scores and compromise comparability across participants. A total of 26 questionnaires (6.58% of all returned forms) were excluded, minimizing the likelihood of significant bias, resulting in a final analytical sample of 369 students, and an overall response rate of 93.4%. Since incomplete questionnaires were excluded before analysis, no imputation methods were applied for missing data. The excluded questionnaires were proportionally distributed across schools and grade levels, indicating that their removal did not materially affect the representativeness of the final sample.

Study tools and measures

Data were collected anonymously using an interviewer-administered questionnaire through one-on-one interviews with each student by the data collectors in a private setting within school premises to ensure confidentiality. Each interview lasted approximately 10-15 minutes. Data collectors underwent two structured training sessions conducted by the research team to ensure consistency and accuracy in questionnaire administration. Training sessions were conducted before data collection to minimize interviewer bias, and periodic supervision was performed throughout the data collection period.

The validated full Arabic version of the questionnaire was obtained with full permission to use and reproduce directly from the original author of a previously published study conducted in Dammam, Saudi Arabia [12]. Minor contextual adaptations (e.g., currency, school grades, grade point average (GPA) levels) were made without altering the original meaning or content.

The questionnaire consisted of five sections.

Section 1 - Sociodemographic Characteristics

This section collected the data on age, nationality, sex, academic grade, school attendance and performance, monthly allowance, leisure activities, parental education and employment status, family income, and gaming status.

Section 2 - Gaming-Related Behavior

This section was completed only by students who reported playing video games (gaming status = Yes). It covered gaming behavior, including the type of games played, device used, age at which gaming began, most frequently played games, time spent playing, and money spent on video games.

Section 3 - Gaming Disorder

IGD was assessed using the nine-item Internet Gaming Disorder Scale-Short Form (IGDS9-SF) [13]. IGDS9-SF consists of nine items corresponding to the DSM-5 diagnostic criteria for IGD, each rated on a five-point Likert scale ranging from 1 (“very rarely”) to 5 (“very often”), with 3 indicating “sometimes”. According to the DSM-5 criteria published by the APA, a diagnosis of IGD requires the endorsement of at least five of the nine criteria. Consistent with previously published studies, participants who responded with a score of 4 (“often”) or 5 (“very often”) on five or more of the nine IGDS9-SF items were classified as having IGD (referred to as IGD 4) [12,14,15]. The Arabic version of the IGDS9-SF used in this study demonstrated acceptable internal consistency, with a Cronbach’s alpha coefficient of 0.769.

Section 4 - Depression Assessment

This section used the Patient Health Questionnaire-9 (PHQ-9) to measure depression severity [16]. PHQ-9 consists of nine items, each scored from 0 (“never”) to 3 (“nearly every day”), resulting in a total score ranging from 0 to 27. Depression severity was categorized as no/minimal (score 0-4), mild (score 5-9), moderate (score 10-14), moderately severe (score 15-19), and severe (score 20-27).

Section 5 - Anxiety Assessment

This section included the Generalized Anxiety Disorder-7 (GAD-7) to assess anxiety severity [17]. Scores ranged from 0 to 21 and were interpreted as no/minimal anxiety (score 0-4), mild anxiety (score 5-9), moderate anxiety (score 10-14), and severe anxiety (score 15-21). The PHQ-9 and GAD-7 instruments were used in their validated Arabic versions, and the cut-off scores were derived from validated Arabic adolescent studies conducted in Saudi Arabia [12,18]. The Arabic versions of PHQ-9 and GAD-7 that were used in this study demonstrated acceptable internal consistency, with a Cronbach’s alpha coefficient of 0.714 and 0.790, respectively.

Full permission was obtained from the original author to use the Arabic version of the Internet Gaming Disorder Scale (IGDS9-SF) and the study questionnaire in this study. The PHQ-9 and GAD-7 are in the public domain and were used in their validated Arabic versions.

Statistical approach

Data evaluation and analysis were performed using R Studio, version 4.2.1 (R Foundation for Statistical Computing, Vienna, Austria). Preliminary data analysis included descriptive statistics, which were presented as frequencies (n) and percentages (%) for categorical variables, and as means with standard deviations (±SDs) for continuous variables. The chi-square test was used to compare categorical variables, while the independent sample t-test was used to compare continuous variables between two independent groups; the threshold of statistical significance for the analytic tests was established at a *P*-value of < 0.05 throughout the analysis.

## Results

Key sociodemographic characteristics of study participants

In this study, a total of 369 high school students from Zawiya City, Libya, participated in the study and completed the survey. Among them, 66.4% of students reported playing video games in the past 12 months (gamers), while 33.6% were non-gamers. Table 1 provides the key sociodemographic characteristics of the total study participants stratified by the gaming status. The mean age of the study participants was 16.2 years (±0.9), and 50.4% were male. Also, 62.4% of gamers were male, compared with 73.4% of non-gamers being female, with significant gender distribution differences (*P* < 0.001). Additionally, 43.1% of the students were in 10th grade (first year, high school), with a statistically significant difference between gamers and non-gamers (*P* = 0.005). Most students (83.2%) reported attending school daily, and the majority had a GPA score of “very good” (41.2%) or “excellent” (37.4%) in their past year grade, with varied academic performance between gamers and non-gamers (*P* = 0.003).

**Table 1:** Demographic Characteristics of Study Participants (n=369), Grouped by Gaming Status LD = Libyan dinar *Statistically significant level of *P*-value <0.05. ^Questions that accepted more than one answer.

Variables	Non-gamers (n=124)	Gamers (n=245)	Test (*P*-value)	Overall (n=369)
Age				
Mean (±SD)	16.3 (±0.9)	16.2 (±0.9)	t=1.64 (0.09)	16.2 (±0.9)
Sex, n (%)				
Male	33 (26.6%)	153 (62.4%)	χ^2^=40.87 (<0.001)*	186 (50.4%)
Female	90 (73.4%)	93 (37.6%)		183 (49.6%)
Academic year, n (%)				
10th grade	43 (35.0%)	116 (47.2%)	χ^2^=10.49 (0.005)*	159 (43.1%)
11th grade	31 (25.2%)	72 (29.3%)		103 (27.9%)
12th grade	49 (39.8%)	58 (23.6%)		107 (29.0%)
School attendance, n (%)				
Every day	111 (89.5%)	196 (80.0%)	χ^2^=7.97 (0.09)	307 (83.2%)
Absences once a week	3 (2.4%)	19 (7.8%)		22 (6.0%)
Absences more than twice a week	2 (1.6%)	12 (4.8%)		14 (3.7%)
Absences once or twice a month	8 (6.5%)	18 (7.3%)		26 (7.0%)
GPA, n (%)				
Excellent	62 (50.0%)	76 (31.0%)	χ^2^=15.65 (0.003)*	138 (37.4%)
Very good	44 (35.5%)	108 (44.1%)		152 (41.2%)
Good	14 (11.3%)	52 (21.2%)		66 (17.9%)
Acceptable or poor	4 (3.2%)	9 (3.6%)		13 (3.5%)
Father's job, n (%)				
Governmental	85 (68.5%)	183 (74.7%)	χ^2^=5.56 (0.2)	268 (72.6%)
Non-governmental	29 (23.4%)	53 (21.6%)		82 (22.2%)
Unemployed	5 (4.0%)	5 (2.0%)		10 (2.7%)
Retired	5 (4.0%)	4 (1.6%)		9 (2.4%)
Father's level of education, n (%)				
Illiterate	8 (6.4%)	16 (6.5%)	χ^2^=4.61 (0.4)	24 (6.5%)
Elementary	3 (2.4%)	11 (4.5%)		14 (3.8%)
Primary	27 (21.8%)	37 (15.1%)		64 (17.3%)
Secondary	32 (25.8%)	78 (31.8%)		110 (29.8%)
University	54 (43.5%)	103 (42.0%)		157 (42.5%)
Mother's job, n (%)				
Governmental	100 (80.6%)	194 (79.2%)	χ^2^=1.18 (0.8)	294 (79.7%)
Non-governmental	4 (3.2%)	8 (3.3%)		12 (3.3%)
Unemployed	18 (14.5%)	38 (15.5%)		56 (15.2%)
Retired	2 (1.6%)	5 (2.0%)		7 (1.8%)
Mother's level of education, n (%)				
Illiterate	1 (0.8%)	1 (0.4%)	χ^2^=1.70 (0.8)	2 (0.5%)
Elementary	2 (1.6%)	8 (3.3%)		10 (2.7%)
Primary	12 (9.7%)	24 (9.8%)		36 (9.8%)
Secondary	30 (24.2%)	54 (22.0%)		84 (22.8%)
University	79 (63.7%)	158 (64.5%)		237 (64.2%)
Family monthly income, n (%)				
<1000 LD	5 (4.1%)	7 (2.8%)	χ^2^=6.30 (0.1)	12 (3.3%)
1000-3000 LD	64 (52.0%)	98 (39.8%)		162 (43.9%)
3000-5000 LD	32 (26.0%)	82 (33.3%)		114 (30.9%)
>5000 LD	4 (3.3%)	20 (8.1%)		24 (6.5%)
Do not know	18 (14.6%)	39 (15.9%)		57 (15.4%)
Student spend per month, n (%)				
<50 LD	32 (26.0%)	48 (19.5%)	χ^2^=2.60 (0.4)	80 (21.7%)
50-100 LD	35 (28.5%)	66 (26.8%)		101 (27.4%)
>100 LD	21 (17.1%)	56 (22.8%)		77 (20.9%)
Unknown	35 (28.5%)	76 (30.9%)		111 (30.1%)
Most common hobbies^, n (%)				
Team sport	25 (13.4%)	121 (28.1%)	χ^2^=47.22 (<0.001)*	146 (23.7%)
Swimming	11 (5.9%)	49 (11.4%)		60 (9.7%)
Drawing	31 (16.7%)	59 (13.7%)		90 (14.6%)
Photography	43 (23.1%)	69 (16%)		112 (18.2%)
Reading	48 (25.8%)	48 (11.1%)		96 (15.6%)
Other sports	6 (3.2%)	37 (8.6%)		43 (6.9%)
Other	14 (7.5%)	30 (7%)		44 (7.1%)
Non-applicable	8 (4.3%)	18 (4.2%)		26 (4.2%)

The majority of students’ parents held government jobs and had university education, 43.9% of families had a monthly income of 1000 to 3000 Libyan dinar (LD), and 27.4% of students spent 50-100 LD monthly. The most common hobbies among gamers were “team sports” (28.1%) and “photography” (16%), whereas non-gamers preferred “reading” (25.8%) and “photography” (23.1%) (*P* < 0.001).

IGD prevalence and gamer characteristics

Among 245 gamers, the mean IGD score was 18.67 (±6.9), ranging from 9 to 39 (Table 2). The prevalence of IGD was 7.3%, based on DSM-5 criteria for IGD. A total of 72.2% of students who met the IGD criteria were male, while 27.8% were female (*P* = 0.5, not significant). With regard to students’ attendance and reported GPA, gamers with IGD had lower school attendance (*P* = 0.002) and lower GPA (*P* = 0.02). It was found that 38.9% of gamers with IGD had a monthly family income of ≤3000 LD, and 44.4% spent >100 LD monthly, with significant income and spending differences (*P* < 0.05).

**Table 2:** Demographic Characteristics of the High School Gamers (n=245) Grouped by IGD Status According to DSM-5 Criteria for IGD IGD = Internet Gaming Disorder, LD = Libyan dinar *Statistically significant level of *P*-value < 0.05.

Variables	Gamers with IGD absent (n=227)	Gamers with IGD present (n=18)	Test (*P*-value)	Overall (n=245)
IGD score				
Mean (±SD)	17.7 (±5.9)	30.9 (±5.7)	t=-9.42 (<0.001)*	18.7 (±6.9)
Age				
Mean (±SD)	16.2 (±0.9)	16.3 (±0.8)	t=-0.54 (0.59)	16.2 (±0.9)
Sex, n (%)				
Male	140 (61.7%)	13 (72.2%)	χ^2^=0.41 (0.52)	153 (62.4%)
Female	87 (38.3%)	5 (27.8%)		92 (37.6%)
Level of education, n (%)				
10th grade	106 (46.7%)	10 (55.6%)	χ^2^=0.60 (0.74)	116 (47.3%)
11th grade	67 (29.5%)	4 (22.2%)		71 (29.0%)
12th grade	54 (23.8%)	4 (22.2%)		58 (23.7%)
School attendance, n (%)				
Every day	202 (89.0%)	12 (66.7%)	χ^2^=16.28 (0.002)*	214 (87.3%)
Absences once a week	17 (7.5%)	2 (11.1%)		19 (7.8%)
Absences more than twice a week	8 (3.5%)	4 (22.2%)		12 (4.9%)
GPA, n (%)				
Excellent	73 (32.2%)	3 (16.6%)	χ^2^=10.73 (0.02)*	76 (31.0%)
Very good	101 (44.5%)	7 (38.9%)		108 (44.0%)
Good	47 (20.7%)	5 (27.8%)		52 (21.2%)
Acceptable or poor	6 (2.6%)	3 (16.6%)		9 (3.6%)
Family monthly income, n (%)				
<1000 LD	7 (3.1%)	0 (0%)	χ^2^=12.10 (0.01)*	7 (2.9%)
1000-3000 LD	91 (40.1%)	7 (38.9%)		98 (40.0%)
3000-5000 LD	77 (33.9%)	5 (27.8%)		82 (33.5%)
>5000 LD	14 (6.2%)	5 (27.8%)		19 (7.8%)
Unknown	38 (16.7%)	1 (5.6%)		39 (15.9%)
Student spend per month, n (%)				
<50 LD	43 (18.9%)	5 (27.8%)	χ^2^=7.95 (0.04)*	48 (19.6%)
50-100 LD	64 (28.2%)	2 (11.1%)		66 (26.9%)
>100 LD	47 (20.7%)	8 (44.4%)		55 (22.4%)
Unknown	73 (32.2%)	3 (16.7%)		76 (31.0%)
Time spent playing games during weekdays, n (%)				
<2 hours a day	123 (54.2%)	6 (33.3%)	χ^2^=10.79 (0.05)*	129 (52.6%)
2-4 hours a day	62 (27.3%)	4 (22.2%)		66 (26.9%)
4-6 hours a day	22 (9.7%)	6 (33.3%)		28 (11.4%)
>6 hours a day	20 (8.8%)	2 (11.1%)		22 (8.9%)
Time spent playing games during weekends, n (%)				
<2 hours a day	73 (32.2%)	1 (5.6%)	χ^2^=13.42 (0.01)*	74 (30.2%)
2-4 hours a day	56 (24.7%)	6 (33.3%)		62 (25.3%)
4-6 hours a day	38 (16.7%)	1 (5.6%)		39 (15.9)
>6 hours a day	60 (26.4%)	10 (55.5%)		70 (28.6%)
Approximate money spent on video games per month, n (%)				
<50 LD	63 (27.8%)	4 (22.2%)	χ^2^=21.49 (<0.001)*	67 (27.3%)
50-100 LD	44 (19.4%)	5 (27.8%)		49 (20.0%)
100-200 LD	7 (3.1%)	4 (22.2%)		11 (4.5%)
200-500 LD	6 (2.6%)	2 (11.1%)		8 (3.3%)
Unknown	107 (47.1%)	3 (16.7%)		110 (44.9%)

Gaming characteristics and behavior among gamers

Regarding the gaming characteristics, gaming duration in hours was significantly higher in students with IGD (*P* < 0.05), both on weekdays and weekends (Table 2). A total of 78% of gamers played online, and 58.3% played video games with friends online (data not shown).

Furthermore, the most frequently played game was PUBG (27%), followed by FIFA (16.3%), with a statistically significant difference in game preference between IGD and non-IGD gamers (*P* = 0.02) (Table 3). Action games (28.5%) were the most popular genres, followed by Adventure (19.9%) and Sport (18.2%) (*P* > 0.05, not significant). In addition, smartphones (61.5%) were the most commonly used gaming device, followed by PlayStation (27%) (*P* > 0.05, not significant).

**Table 3:** Gaming Characteristics Based on Game Name, Genres, and Gaming Devices Among Gamers (n=245) Grouped by IGD Status IGD = Internet Gaming Disorder *Statistically significant level of *P*-value ≤ 0.05. ^Questions that accepted more than one answer.

	Gamers with IGD absent	Gamers with IGD present	Test (*P*-value)	Overall
Most common games played by gamers^ (n=245)				
Assassin's Creed	10 (2.2%)	2 (3.8%)	χ^2^=26.74 (0.02)*	12 (2.4%)
Call of Duty	32 (7.1%)	3 (5.7%)		35 (6.9%)
Clash of Clans	32 (7.1%)	3 (5.7%)		35 (6.9%)
Clash Royale	13 (2.9%)	1 (1.9%)		14 (2.8%)
FIFA	75 (16.6%)	7 (13.2%)		82 (16.3%)
Fortnite	14 (3.1%)	3 (5.7%)		17 (3.4%)
Free Fire	22 (4.9%)	3 (5.7%)		25 (5.0%)
League of Legends	9 (2.0%)	1 (1.9%)		10 (2.0%)
Minecraft	25 (5.5%)	3 (5.7%)		28 (5.6%)
PUBG	123 (27.3%)	13 (24.5%)		136 (27.0%)
Rocket League	4 (0.9%)	2 (3.8%)		6 (1.2%)
Other	87 (19.2%)	12 (22.6%)		99 (19.6%)
Most common video game genres^ (n=245)				
Role-playing game (RPG)	17 (4.0%)	3 (6.8%)	χ^2^=4.92 (0.8)	20 (4.2%)
Action	123 (28.7%)	12 (27.3%)		135 (28.5%)
Adventure	84 (19.6%)	10 (22.7%)		94 (19.9%)
Sport	78 (18.2%)	8 (18.2%)		86 (18.2%)
Shooter	48 (11.2%)	6 (13.6%)		54 (11.4%)
Open World	46 (10.7%)	3 (6.8%)		49 (10.4%)
Horror	10 (2.3%)	2 (4.5%)		12 (2.5%)
Other	23 (5.3%)	0 (0%)		23 (4.8%)
Device/gadget used to play^ (n=245)				
PlayStation	85 (27.1%)	8 (27.6%)	χ^2^=2.45 (0.7)	93 (27%)
Xbox	8 (2.5%)	0 (0%)		8 (2.3%)
Personal computer	12 (3.8%)	1 (3.4%)		13 (3.8%)
Laptop	9 (2.9%)	2 (6.9%)		11 (3.2%)
Smartphone	194 (61.8%)	17 (58.6%)		211 (61.5%)
Tablet	6 (1.9%)	1 (3.4%)		7 (2.0%)

Anxiety and depression among gamers

**Figure 1:**
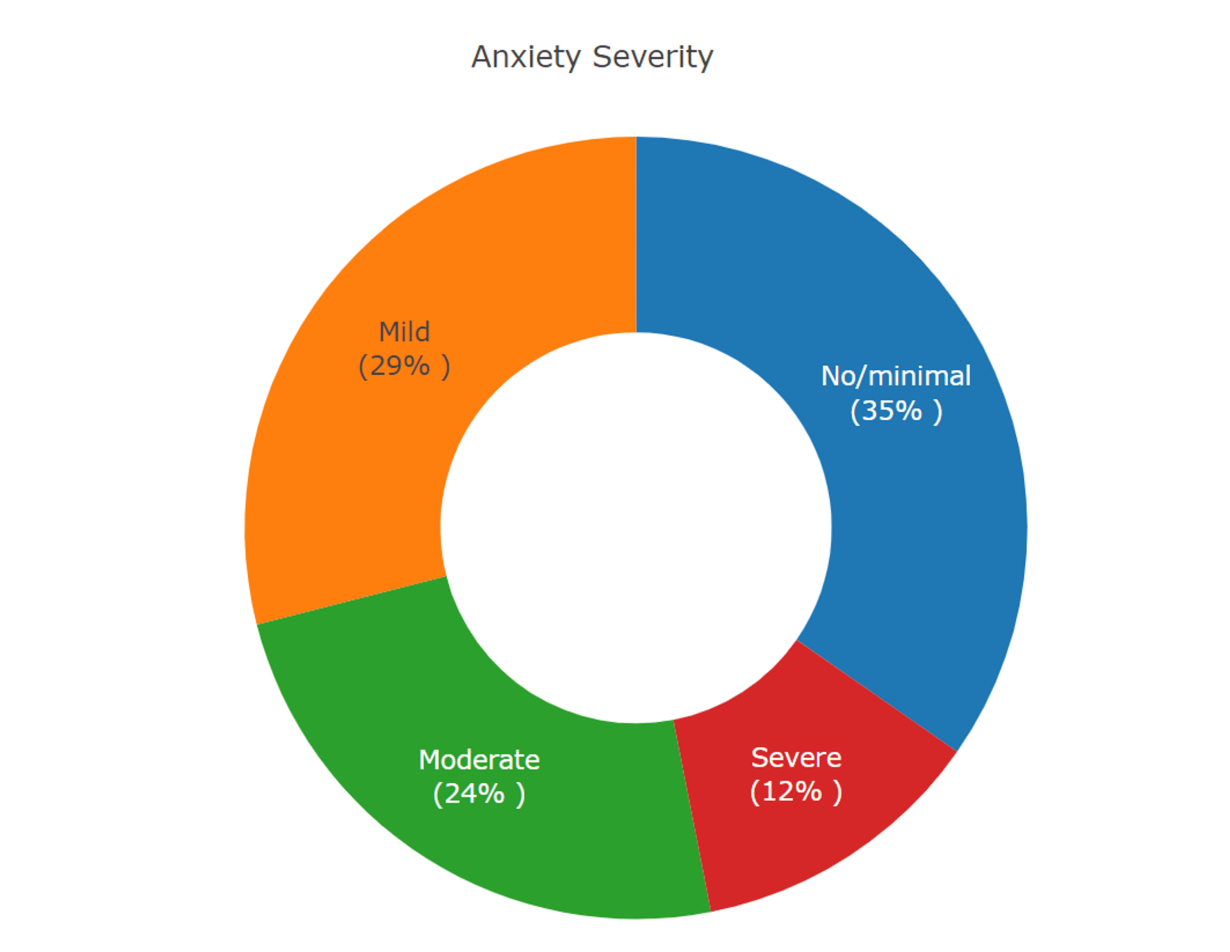
Prevalence of Anxiety Severity Levels This pie chart illustrates the percentage of high school gamers (n=245) within each anxiety severity category.

**Figure 2:**
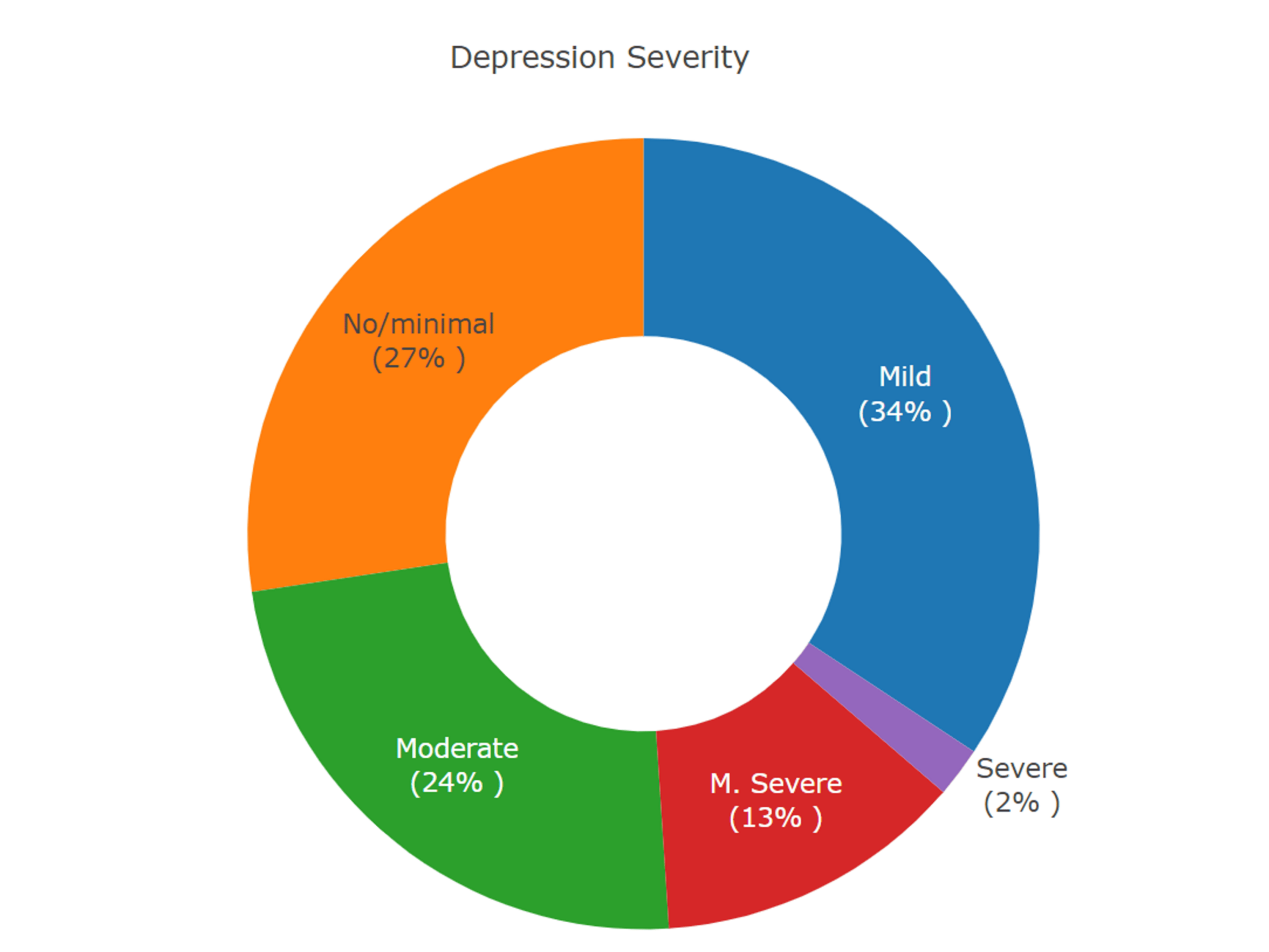
Prevalence of Depression Severity Levels This pie chart illustrates the percentage of high school gamers (n=245) within each depression category: no or minimal depression, mild, moderate, moderately severe (M. severe), and severe.

The mean anxiety score among gamers was 7.66 (±5.1) with no significant differences based on IGD status (*P* = 0.3) (Table 4). However, the mean depression score was significantly higher in students with IGD (11.4 vs. 8.1, *P* = 0.02). More than half of gamers had mild to moderate anxiety, while 12% had severe anxiety (Figure 1). Based on IGD status, gamers with IGD had significantly higher levels of moderate-to-severe anxiety, compared to non-IGD gamers (*P* = 0.02) (Table 4). In relation to depression severity, more than half of all gamers had mild to moderate depression (Figure 2). Based on IGD status, around 14% of non-IGD gamers and 28% of IGD gamers had moderately severe to severe depression, though no significant statistical differences were seen (*P* = 0.12) (Table 4).

**Table 4:** Distribution of Anxiety and Depression Severity Levels Among Gamers (n=245) Grouped by IGD Status IGD = Internet Gaming Disorder *Statistically significant level of *P*-value ≤ 0.05.

	Gamers with IGD absent (n=227)	Gamers with IGD present (n=18)	Test (*P*-value)
Anxiety score, mean (±SD)	7.56 (±5.0)	8.8 (±5.1)	t=-0.99 (0.3)
Depression score, mean (±SD)	8.1 (±5.0)	11.4 (±5.2)	t=-2.53 (0.02)*
Anxiety category			
No/minimal	79 (34.8%)	6 (33.3%)	χ^2^=9.13 (0.02)*
Mild	70 (30.8%)	1 (5.6%)	
Moderate	50 (22.0%)	9 (50.0%)	
Severe	28 (12.3%)	2 (11.1%)	
Depression category			
No/minimal	65 (28.6%)	2 (11.1%)	χ^2^=7.18 (0.12)
Mild	80 (35.2%)	4 (22.2%)	
Moderate	51 (22.5%)	7 (38.9%)	
Moderately severe	27 (11.9%)	4 (22.2%)	
Severe	4 (1.8%)	1 (5.6%)	

## Discussion

This study assessed the prevalence of IGD and its associated sociodemographic, behavioral, academic, and psychosocial factors among adolescents in Zawiya City, Libya. Of a sample of 369 high school students, 245 were classified as internet gamers. Based on the DSM-5 diagnostic criteria, the prevalence of IGD in this gamer group was 7.3%. This estimate was generally in line with recent global epidemiological models, which have reported prevalence estimates ranging from 1% to 10%, depending on the heterogeneity of the studied population, cultural context (e.g., social acceptability of gaming), and study design, with differences in the level of diagnostic thresholds [19]. Stevens et al. (2021), for example, found a global average prevalence of 3.05% in their report, and also reported variation depending on geographical location (e.g., South Korea had a higher average prevalence of 5.9% in adolescents compared to Western countries, where the rate was ~1.6% [4,20,21]. The high prevalence (7.3%) may also reflect concurrent influences related to cultural aspects of gaming, increased socioeconomic stress that motivates gaming behavior, or variations in sampling methods (e.g., in the selection of subjects from gaming-specific locations). The variation in estimates suggests that IGD should be interpreted within its local sociocultural context, particularly considering environmental and behavioral factors that may influence gaming behaviors.

The findings for IGD gamers showed that 72.2% were male and 27.8% were female gamers, which is consistent with international meta-analyses suggesting that males are significantly more likely to develop IGD than females [22]. For instance, a recent study of Slovenian adolescents found that nearly all IGD gamers were male (87.5%), and another investigation in Brazil found that 57.5% of those meeting the DSM-5 criteria for IGD were male [14,23]. Although no statistically significant differences in IGD status were observed across gender in this study, future research may further explore gender-related patterns in gaming behavior and their potential association with IGD, particularly within different sociocultural contexts.

Gamers with IGD exhibited statistically significant reductions in both school attendance (*P* = 0.002) and GPA scores (*P* = 0.02). Previous research has shown that increased gaming usage correlates with decreased academic performance. For instance, a study examining the effects of online gaming addiction among Chinese university students found that those suffering from gaming addiction displayed lower academic performance, attributed to the adverse effects of gaming on their behavioral, emotional, and cognitive engagement [24]. In this study, IGD was associated with lower academic performance indicators, suggesting a potential relationship between gaming behavior and educational outcomes among adolescents. The immersive nature of gaming may be linked to coping patterns that divert attention from academic responsibilities; however, cautious interpretation should be considered in light of the study’s cross-sectional design.

Among IGD gamers, 38.9% were from households with a monthly income of less than 3000 LD, while 44.4% reported spending over 100 LD on gaming-related expenses. In addition, IGD status was significantly associated with socioeconomic status (SES) and with students’ monthly video game expenditures (*P* < 0.05). These findings align with the research conducted by Mihara and Higuchi (2017), which indicated a relationship between lower SES in Japan and an increased risk of IGD, suggesting that limited access to alternative leisure activities and potential psychosocial triggers may serve as mediating factors [25]. Furthermore, the observed relationship between gaming expenditures and IGD aligns with behavioral financial theories, which suggest that economic commitments can reinforce habitual behaviors and may contribute to the persistence or escalation of compulsive gaming. The association between SES and IGD warrants further investigation, particularly given the unclear directionality of this relationship. For example, lower income may be associated with increased engagement in escapist gaming behaviors, while IGD may also contribute to economic strain through financial costs and reduced productivity.

Our study findings highlight the association between IGD and mental health outcomes among adolescent gamers. Although the mean anxiety scores did not differ significantly between students with or without IGD (*P* = 0.3), the distribution of severity illustrated a different pattern: students with IGD were significantly more likely to experience moderate-to-severe anxiety (*P* = 0.02). This suggests that while the overall burden of anxiety may appear similar between groups, clinically meaningful severity is disproportionately higher among those with IGD. With regard to depression, a statistically significant difference in mean scores was observed (11.4 for IGD vs. 8.1 for non-IGD, *P* = 0.02), along with a higher proportion of moderate-to-severe depression symptoms in the IGD group (28% vs. 14%), although this latter difference did not reach statistical significance (*P* = 0.12). These findings are consistent with previous international studies reporting moderate-to-strong associations between IGD and depression symptoms [26-28]. In contrast, the weaker association between IGD and anxiety observed in this study, compared with some global reports, may reflect cultural differences in symptom expression, measurement approaches, or sample characteristics; for instance, student populations may exhibit baseline anxiety due to academic pressures, which would obscure group differences [29,30]. Overall, these findings suggest that even when some mental health measures do not show statistically significant group differences, clinically relevant increases in symptom severity, particularly for depression and acute anxiety, may still be observed among adolescents with IGD, highlighting the potential importance of targeted screening and early intervention strategies.

In summary, these findings highlight the interplay between sociodemographic, behavioral, and psychological factors and IGD, underscoring its relevance as a complex public health concern among adolescents. This study has several strengths. It represents one of the first epidemiological investigations of IGD among high school students in Libya, addressing a notable regional research gap. The use of multistage cluster sampling enhances representativeness within the selected population. In addition, standardized and widely validated instruments (IGDS9-SF, PHQ-9, and GAD-7) were employed, supporting measurement reliability and comparability with international research. Despite these strengths, several limitations should be considered. The cross-sectional design precludes establishing causality between IGD and associated psychological, sociodemographic, academic, and behavioral factors. The study population was restricted to public high school students in a single city, which may limit the generalizability of the findings to adolescents in other regions or educational settings. Reliance on self-reported measures introduces the possibility of recall bias, misclassification, and social desirability bias. Additionally, unmeasured confounding factors may have influenced the observed association. Despite these limitations, the findings provide important preliminary evidence to guide future longitudinal and intervention-based research.

These findings highlight the potential importance of culturally relevant and context-specific public health strategies, while underscoring the need for longitudinal and multicenter studies to better understand the temporal and causal relationships underlying IGD.

## Conclusions

This study contributes to the limited body of evidence on IGD within the Libyan context and highlights the importance of multidisciplinary research that integrates clinical, sociological, and economic perspectives. The findings are consistent with the global literature demonstrating a strong association between IGD and depression symptoms. In contrast, the weaker association observed between IGD and anxiety in this study may reflect cultural, methodological, or sample-specific factors, including academic demands and contextual stressors unique to student populations. Nevertheless, the presence of elevated levels of depression and acute anxiety among adolescents with IGD underscores the potential importance of early screening and the development of culturally sensitive, targeted interventions for at-risk groups. Future research should seek to clarify the underlying mechanisms linking IGD to mental health outcomes, particularly the role of socioeconomic factors, and to inform the design of effective prevention and intervention strategies that address these complex and context-dependent relationships.

## Appendices

The questionnaire below reflects the instruments administered to participants. Arabic versions were used for data collection, and English versions are provided here for reference and replication. The questionnaire was adapted from a previously published study [12] distributed under the Creative Commons Attribution-Noncommercial-Sharealike 4.0 license, which permits reuse and adaptation with appropriate attribution.

Appendix A: Study questionnaire (English version)

**Table 5:** English Version of the Study Questionnaire LD = Libyan dinar

Section 1: Sociodemographic Information		
Item no.	Question	Response options
1	Nationality	Open-ended
2	Age	Open-ended
3	School name	Open-ended
4	Academic year	10th grade; 11th grade; 12th grade
5	Sex	Male; female
6	Average school attendance	Every day; absences once a week; absence 2 or 3 times a week; absence more than 3 times a week; absence once or twice a month
7	GPA	Poor; acceptable; good; very good; excellent
8	Did you repeat one of the school years?	Yes; no
9	Are you a newcomer in this school year?	Yes; no
10	Do you work a part-time job?	Yes; no
11	Father level of education	Illiterate; elementary; primary; secondary; university; don’t know
12	Mother level of education	Illiterate; elementary; primary; secondary; university; don’t know
13	Father job	Governmental; non-governmental; unemployed; retired; other
14	Mother job	Housewife; governmental; non-governmental; retired; other
15	Family monthly income (LD)	Less than 1000; 1000-3000; 3000-5000; more than 5000; don’t know
16	Monthly allowance/spending money	Less than 50; 50-100; more than 100; unknown
17	Hobbies (multiple choice)	Team sport; swimming; drawing; photography; reading; other sports; non-applicable; others
18	Have you played video games in the last 12 months?	Yes; no
Section 2: Gaming Behavior		
Item no.	Question	Response options
1	Daily gaming hours (weekdays)	<2 hours; 2-4 hours; 4-6 hours; 6-8 hours; >8 hours
2	Daily gaming hours (weekends)	<2 hours; 2-4 hours; 4-6 hours; 6-8 hours; >8 hours
3	Type of gaming device (multiple choice)	PlayStation; Xbox; Nintendo; PC; laptop; smartphone; tablet; others
4	Social context (who do you play with?)	Alone; friends online; friends in-person; others; N/A
5	Game spent most time on recently	Open-ended
6	Online vs. offline	Online; offline
7	Game genre (multiple choice)	RPG; action; adventure; sport; shooter; open world; horror; others
8	Money spent on video games per month	<50 LD; 50-100 LD; 100-200 LD; 200-500 LD; N/A
9	Age started playing video games	<7 years; 7-10 years; 10-15 years; >15 years
Section 3: Internet Gaming Disorder Scale-9 Short Form (IGDS9-SF), response scale: never, rarely, sometimes, often, very often		
Item no.	Item text	
1	Do you feel preoccupied with your gaming behavior? (Thinking about previous sessions, anticipating the next, etc.)	
2	Do you feel more irritability, anxiety, or even sadness when you try to either reduce or stop your gaming activity?	
3	Do you feel the need to spend increasing amount of time engaged gaming in order to achieve satisfaction or pleasure?	
4	Do you systematically fail when trying to control or cease your gaming activity?	
5	Have you lost interests in previous hobbies and other entertainment activities as a result of your engagement with the game?	
6	Have you continued your gaming activity despite knowing it was causing problems between you and other people?	
7	Have you deceived any of your family members, friends, or others because the amount of your gaming activity?	
8	Do you play in order to temporarily escape or relieve a negative mood (e.g., helplessness, guilt, anxiety)?	
9	Have you jeopardized or lost an important relationship, job, or an educational or career opportunity because of your gaming?	
Section 4: Patient Health Questionnaire-9 (PHQ-9), response scale: not at all, several days, more than half the days, nearly every day		
Item no.	Statement	
1	Little interest or pleasure in doing things	
2	Feeling down, depressed, or hopeless	
3	Trouble falling or staying asleep, or sleeping too much	
4	Feeling tired or having little energy	
5	Poor appetite or overeating	
6	Feeling bad about yourself - or that you are a failure or you have let yourself or your family down	
7	Trouble concentrating on things, such as reading the newspaper or watching television	
8	Moving or speaking so slowly that other people could have noticed or the opposite - being so fidgety or restless	
9	Thought that you would be better off dead or of hurting yourself in some way	
Section 5: Generalized Anxiety Disorder-7 (GAD-7), response scale: not at all, several days, more than half the days, nearly every day		
Item no.	Statement	
1	Feeling nervous, anxious or on edge	
2	Not being able to stop or control worrying	
3	Worrying too much about different things	
4	Trouble relaxing	
5	Being so restless that it is hard to sit still	
6	Becoming easily annoyed or irritable	
7	Feeling afraid as if something awful might happen	

Appendix B: Study questionnaire (Arabic version)

**Table 6:** Arabic Version of the Study Questionnaire

القسم الأول: البيانات الديموغرافية والاجتماعية		
خيارات الإجابة	السؤال	رقم الفقرة
إجابة مفتوحة	الجنسية	1
إجابة مفتوحة	العمر (لأقرب سنة)	2
إجابة مفتوحة	اسم المدرسة	3
أولى ثانوي؛ ثانية ثانوي؛ ثالثة ثانوي	المرحلة الدراسية	4
ذكر؛ أنثى	الجنس	5
احضر بشكل يومي؛ اتغيب مرة في الاسبوع؛ اتغيب من مرتين الى ثلاث في الاسبوع؛ اتغيب اكثر من ثلاث مرات في الاسبوع؛ اتغيب مرة او مرتين في الشهر	حضورك المدرسة خلال الأشهر القليلة الماضية	6
ضعيف؛ مقبول؛ جيد؛ جيد جدا؛ ممتاز	المعدل الدراسي الحالي	7
نعم؛ لا	هل أعدت احد السنوات الدراسية؟	8
نعم؛ لا	هل انت مستجد في هذه السنة الدراسية؟	9
نعم؛ لا	هل تعمل بوظيفة بدوام جزئي؟	10
لا يقرأ ولا يكتب؛ إبتدائي؛ متوسط؛ ثانوي أو دبلوم؛ جامعي؛ لا أعلم	مستوى تعليم الأب	11
لا تقرأ ولا تكتب؛ إبتدائي؛ متوسط؛ ثانوي أو دبلوم؛ جامعي؛ لا أعلم	مستوى تعليم الأم	12
قطاع العام؛ عمل حر؛ لا يعمل حاليا؛ متقاعد؛ أخرى	وظيفة الأب	13
ربة منزل متفرغة؛ قطاع العام؛ عمل حر؛ متقاعدة؛ أخرى	وظيفة الأم	14
أقل من 1000؛ من 1000 إلى 3000؛ من 3000 إلى 5000؛ أكثر من 5000؛ لا أعلم	دخل الأسرة الشهري (بالدينار الليبي)	15
أقل من 50؛ من 50 إلى 100؛ أكثر من 100؛ غير محدد	مصروفك الشهري	16
رياضة جماعية؛ سباحة؛ رسم؛ تصوير؛ قراءة؛ ألعاب رياضية أخرى؛ لا ينطبق؛ أخرى	الهوايات (يمكن اختيار أكثر من إجابة)	17
نعم؛ لا	هل لعبت ألعاب إلكترونية خلال الـ 12 شهر الماضية؟	18
القسم الثاني: سلوك اللعب		
خيارات الإجابة	السؤال	رقم الفقرة
أقل من ساعتين؛ 2-4 ساعات؛ 4-6 ساعات؛ 6-8 ساعات؛ أكثر من 8 ساعات	ساعات اللعب في اليوم خلال أيام الأسبوع (الأحد إلى الخميس)	1
أقل من ساعتين؛ 2-4 ساعات؛ 4-6 ساعات؛ 6-8 ساعات؛ أكثر من 8 ساعات	ساعات اللعب خلال أيام عطلة نهاية الأسبوع (الجمعة والسبت)	2
سوني بلايستيشن؛ إكسبوكس؛ نينتيندو؛ كمبيوتر شخصي؛ كمبيوتر محمول (لابتوب)؛ هاتف نقال؛ جهاز لوحي؛ أخرى	نوع جهاز اللعب (يمكن اختيار أكثر من إجابة)	3
لوحدك؛ مع أصدقائك عبر الإنترنت؛ مع أصدقائك على نفس الجهاز؛ آخر؛ لا ينطبق	مع من تلعب عادةً؟	4
إجابة مفتوحة	أكثر لعبة تقضي فيها وقتك حالياً	5
متصل بالإنترنت (أونلاين)؛ غير متصل (أوفلاين)	نوع اللعب الغالب	6
تعدد أدوار (RPG)؛ اكشن؛ مغامرة؛ رياضة؛ قتال؛ عالم مفتوح؛ رعب؛ أخرى	تصنيف اللعبة (يمكن اختيار أكثر من إجابة)	7
أقل من 50 دينار؛ 50-100 دينار؛ 100-200 دينار؛ 200-500 دينار؛ لا ينطبق	المبالغ المالية المنفقة شهرياً على الألعاب	8
أقل من 7 سنوات؛ 7-10 سنوات؛ 10-15 سنة؛ أكثر من 15 سنة	العمر عند بدء ممارسة الألعاب الإلكترونية	9
(IGDS9-SF) القسم الثالث: مقياس اضطراب ألعاب الإنترنت النسخة القصيرة مقياس الإجابة: أبدًا، نادرًا، أحيانًا، كثيرًا، كثيرًا جدًا		
نص السؤال		رقم الفقرة
هل تشعر بأنك مشغول البال بسبب الألعاب؟ (التفكير في الجلسات السابقة، انتظار الجلسة القادمة، إلخ)		1
هل تشعر بالانزعاج، القلق، أو الحزن، عند محاولة التقليل أو التوقف عن نشاطات اللعب؟		2
هل تشعر بالحاجة إلى زيادة الوقت المنفق في اللعب لكي تحصل على الرضا التام أو المتعة؟		3
هل تفشل مخططاتك عندما تحاول التحكم أو التوقف عن اللعب؟		4
هل فقدت اهتماماتك أو هواياتك السابقة أو الأنشطة الترفيهية الأخرى كنتيجة لانشغالك باللعب؟		5
هل واصلت نشاطات اللعب على الرغم من معرفتك بأنها تسبب لك المشاكل مع الآخرين؟		6
هل كذبت قط على عائلتك أو أصحابك أو غيرهم عن عدد الساعات التي تقضيها باللعب؟		7
هل تلعب من أجل الهروب مؤقتًا أو التخفيف من المزاج السلبي (القلق، الشعور بالذنب، الحزن، إلخ)؟		8
هل خاطرت أو خسرت علاقة، وظيفة، معدلاً دراسيًا، أو فرصة مهنية بسبب نشاطك في اللعب؟		9
القسم الرابع: استبيان صحة المريض(PHQ-9) مقياس الإجابة: أبدًا، بعض الأيام، أكثر من نصف الأيام، كل يوم تقريبًا		
المشكلة		رقم الفقرة
قلة الاهتمام أو الاستمتاع بممارسة الأشياء.		1
الشعور بالحزن أو ضيق الصدر أو اليأس.		2
الصعوبة في الركون إلى النوم أو النوم بانتظام أو النوم أكثر من العادة.		3
الشعور بالتعب أو بقلة الحيوية.		4
قلة الشهية أو كثرة الأكل.		5
الشعور بعدم الرضا عن النفس أو بالفشل أو الإحباط تجاه ذويك.		6
الصعوبة في التركيز على الأشياء، مثل قراءة الصحف أو مشاهدة التلفزيون.		7
بطء في الحركة أو الكلام أو العكس كثرة التملل والتحرك بدرجة ملحوظة.		8
التفكير بإيذاء النفس بطريقة ما.		9
القسم الخامس: مقياس اضطراب القلق العام (GAD-7) مقياس الإجابة: أبدًا، بعض الأيام، أكثر من نصف الأيام، كل يوم تقريبًا		
المشكلة		رقم الفقرة
الشعور بالغضب أو القلق أو الانفعال الشديد.		1
عدم القدرة على إنهاء القلق أو التحكّم فيه.		2
القلق المفرط على أشياء مختلفة.		3
الصعوبة في الاسترخاء.		4
شدة الاضطراب لدرجة صعوبة البقاء في هدوء.		5
السرعة في الانزعاج أو الانفعال.		6
الشّعور بالخوف كما لو أن شيئًا فظيعًا قد يحدث.		7
